# Advanced Oxidation Nanoprocessing Boosts Immunogenicity of Whole Tumor Cells

**DOI:** 10.1002/advs.202302250

**Published:** 2023-05-21

**Authors:** Min Zhang, Yiming Huang, Jie Zou, Yang Yang, Yue Yao, Guofeng Cheng, Yannan Yang

**Affiliations:** ^1^ Clinical Medicine Scientific and Technical Innovation Center Shanghai Tenth People's Hospital Tongji University School of Medicine Shanghai 200092 P. R. China; ^2^ Key Laboratory of Colloid and Interface Chemistry of the Ministry of Education School of Chemistry and Chemical Engineering Shandong University Jinan Shandong 250100 P. R. China; ^3^ Shanghai Frontiers Science Research Base of Intelligent Optoelectronics and Perception, Institute of Optoelectronics Fudan University Shanghai 200433 P. R. China; ^4^ Australian Institute for Bioengineering and Nanotechnology The University of Queensland Brisbane Queensland 4072 Australia

**Keywords:** advanced oxidation, immunogenic apoptosis, nano–bio interfaces, whole tumor antigens

## Abstract

Whole tumor cells expressing a wide array of tumor antigens are considered as a highly promising source of antigens for cancer vaccines. However, simultaneously preserving the antigen diversity, improving immunogenicity, and eliminating the potential tumorigenic risk of whole tumor cells are highly challenging. Inspired by the recent progress in sulfate radical‐based environmental technology, herein, an advanced oxidation nanoprocessing (AONP) strategy is developed for boosting the immunogenicity of whole tumor cells. The AONP is based on the activation of peroxymonosulfate by ZIF‐67 nanocatalysts to produce SO_4_^−∙^ radicals continuously, leading to sustained oxidative damage to tumor cells and consequently extensive cell death. Importantly, AONP causes immunogenic apoptosis as evidenced by the release of a series of characteristic damage associated molecular patterns and at the same time maintains the integrity of cancer cells, which is critical to preserve the cellular components and thus maximize the diversity of antigens. Finally, the immunogenicity of AONP‐treated whole tumor cells is evaluated in a prophylactic vaccination model, demonstrating significantly delayed tumor growth and increased survival rate of live tumor‐cell‐challenged mice. It is expected that the developed AONP strategy would pave the way to develop effective personalized whole tumor cell vaccines in future.

## Graphical abstract

Inspired by the advanced oxidation in environment applications, this work presents a facile and robust advanced oxidation nanoprocessing (AONP) strategy for boosting the immunogenicity of whole tumor cells. AONP is realized by simply mixing peroxymonosulfate and ZIF‐67 nanoparticles, which produce massive SO_4_^−∙^ radicals to potently induce immunogenic apoptosis of tumor cells, thus converting them into antigens to elicit antitumor immunity.

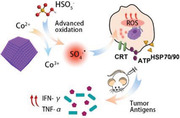

## 1 Introduction

Even many efforts have been devoted to discover tumor antigens, the development of cancer vaccines and their clinical efficacy is still hindered.^[^ ^1^ ^]^ A major reason is that, for most types of cancers, the number of available tumor‐associated antigens (TAAs) to construct vaccines is very limited. Moreover, the immunogenicity of TAAs is also limited, and is highly variable among individuals.^[^ ^2^ ^]^ Compared to TAAs, tumor‐specific antigens (TSAs) have been proven effective in provoking stronger antitumor immunity, but the difficulties in both scientific and technical aspects, including TSA identification, fabrication, and validation, largely impede their usage in clinics.^[^ ^3^ ^]^ Distinct from strategies that target single antigens, whole tumor cells expressing a wide array of tumor antigens have been considered highly promising sources of antigens for cancer vaccines, which can potentially overcome the low immunogenicity of TAAs and the time‐consuming and complicated fabrication process of TSAs, and thus have attracted tremendous research efforts.^[^ ^4^ ^]^

Currently, ultraviolet B (UVB) ray irradiation and repeat cycles of freezing and thawing are the two most commonly used methods in the clinic to prepare whole tumor cell antigens.^[^ ^5^ ^]^ UVB irradiation induces apoptosis, leading to the exposure of a range of “eat me” signals on the tumor cell surface to facilitate phagocytosis by antigen‐presenting cells. However, the limited immunogenicity of apoptotic cells induces by UVB irradiation results in unsatisfactory immunogenicity of whole tumor cells, and the uneven dose of UVB irradiation could potentially cause tumorigenesis upon vaccination.^[^ ^5^, ^6^ ^]^ Repeat freezing and thawing treatment can result in necrosis of tumor cells, producing cell lysates that contain cellular components to trigger the immune response. However, the use of fragmented cells instead of whole components of tumor cells may lead to compromised immunogenicity due to the inevitable loss of a large portion of tumor antigens.^[^ ^5^, ^7^ ^]^ Hence, developing an alternative robust treatment approach that could simultaneously 1) maintaining integrity and components, 2) eliminating tumorigenic risk, and 3) promoting immunogenicity of whole tumor cells is considered of great promise for clinic translation, yet highly challenging.

As an innovative environmental technology for water remediation, advanced oxidation processes (AOP) exhibited superior oxidative degradation performance toward versatile organic pollutants than conventional hydroxyl radical‐based Fenton processes. This is due mainly to the unique advantages of sulfur‐based radicals (e.g., SO_4_^ˉ∙^) compared to conventional oxygen‐based radical (e.g., OH∙), including increased redox potential (2.5−3.1 V), longer half‐life (30–40 µs) and higher selectivity toward organic materials.^[^ ^8^ ^]^ Coincidently, organic pollutants and tumor cells share two key similarities: both are essentially organic materials, and both are surrounded by an aqueous environment. Given these similarities in organic pollutants and tumor cells, as well as the capability of oxidative damage in promoting significant cell death and immunogenicity,^[^ ^9^ ^]^ we speculate that the AOP that normally used in environmental technologies can be potentially applied in biomedical field, particularly for fabricating whole tumor cell lysates and boosting their immunogenicity. In addition, it has been well‐documented that nanomaterials can greatly benefit the catalytic performance of AOP via efficient and sustained SO_4_^−∙^ production,^[^ ^10^ ^]^ which is expected to further improve the AOP‐based cell treatment strategy.

**Figure 1:**
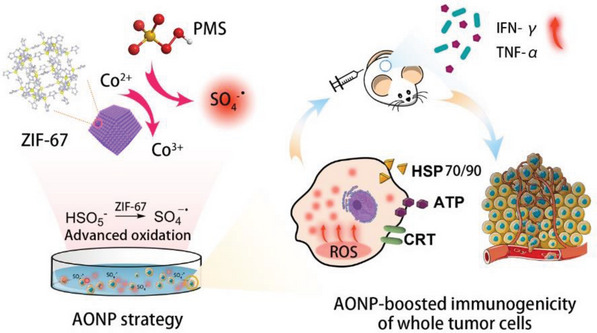
Schematic illustration of advanced oxidation treated whole tumor cells with boosted immunogenicity eliciting efficient antitumor immune response.

Herein, a novel advanced oxidation nanoprocessing (AONP) strategy is developed to boost the immunogenicity of whole tumor cells. The AONP system contains peroxymonosulfate (PMS), a commercially available and cheap precursor of SO_4_^−∙^, and ZIF‐67 nanoparticles as a heterogeneous cobalt catalyst to activate PMS. As illustrated in **Figure** **1**, the AONP based on the mixture of PMS and ZIF‐67 nanoparticles could continuously produce SO_4_^−∙^ radicals, which led to massive oxidative damage to cancer cells and consequently immunogenic apoptosis. AONP not only maintained cells’ integrity and components, but also caused the release of a wide spectrum of DAMPs, including CRT, HMGB1, HSP70/90, and ATP. The AONP‐enhanced immunogenicity of whole tumor antigens was further demonstrated in a prophylactic vaccination model in vivo, showing significantly delayed tumor growth profile and survival rate of tumor‐bearing mice.

## 2 Results and Discussion

**Figure 2:**
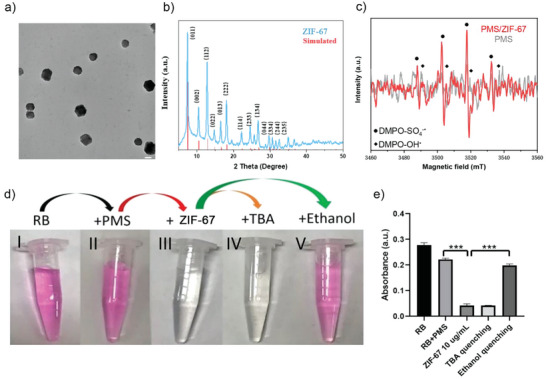
Construction and oxidative activity of AONP. a) TEM image of ZIF‐67 nanoparticles after PEG modification. Scale bar: 100 nm. b) XRD pattern of ZIF‐67 nanoparticles. c) EPR spectrum of PMS and PMS activated by ZIF‐67. d,e) Degradation of Rhodamine B due to OH∙ and SO_4_^−∙^ generation: I) control, II) tube I with 150 µg mL^−1^ PMS, III) tube II with 10 µg mL^−1^ ZIF‐67, IV) tube III with quencher tetra‐butanol (TBA), V) tube III with quencher ethanol (*n* = 3). Statistics: e) one‐way ANOVA with Bonferroni's correction for multiple comparisons. Two‐sided test was conducted to determine whether there is significant difference between the mean value of variable *X* and the population mean. Data were shown as mean ± SEM. *p < 0.05, **p < 0.01, ***p < 0.001, ****p < 0.0001; ns: no significant difference.

ZIF‐67 nanoparticles were fabricated following an reported method.^[^ ^11^ ^]^ Owing to the hydrophobicity of ZIF‐67 nanoparticles, the surface was further modified with PEG toward monodispersion in aqueous solution. The morphology of modified sample was characterized by TEM (**Figure** **2**a; Figure S1, Supporting Information), monodispersed nanoparticles with particle size approximate 150 nm can be observed, which is in agreement with Dynamic Light Scattering size distribution curves (160.6 nm) as shown in Figure S2 (Supporting Information). The crystalline structure of as‐prepared ZIF‐67 nanoparticles was further validated by X‐ray diffraction (XRD), displaying characteristic peaks that assigned to ZIF‐67 were well indexed (Figure 2b). Furthermore, the valence state of Co from ZIF‐67 was investigated by X‐ray photoelectron spectroscopy. As shown in Figure S3a (Supporting Information), The Co 2p_3/2_ peak could be deconvoluted into two components corresponding to Co^2+^ (782.9 eV) and Co^3+^ (781.4 eV), which is in agreement with spectrum of typical ZIF‐67.^[^ ^12^ ^]^ To examine whether ZIF‐67 could activate PMS to generate SO_4_^−∙^, electron paramagnetic resonance (EPR) test was performed by mixing ZIF‐67 nanoparticles and PMS in 5,5‐dimethyl‐1‐pirroline‐N‐oxide (DMPO) (a spin‐trapping reagent) aqueous solution. As shown in Figure 2c, peaks assigned to DMPO‐SO_4_^−∙^ could be well identified, confirming the successful generation of SO_4_^−∙^ by PMS/ZIF‐67. In contrast, the generation of SO_4_^−∙^ is much weaker in the absence of ZIF‐67. In addition, hydroxy radicals (OH∙) were also detected in this system, which is in good agreement with previous reports.^[^ ^13^ ^]^ High resolution XPS spectrum suggest that Co^2+^ weights 64.8% in the as‐prepared ZIF‐67 nanoparticles, whereas the proportion drop to 40.3% after PMS activation, suggesting a significant transformation of Co^2+^ to Co^3+^ (Figure S3, Supporting Information). Thus, it can be concluded that PMS was activated by ZIF‐67 following Fenton type reaction.^[^ ^14^ ^]^

The oxidative capability of AONP was examined by incubating PMS/ZIF‐67 with Rhodamine B (RB, a dye indicator) which can be oxidized into colorless by oxidative radicals. As shown in Figure 2d, RB aqueous solution (Tube I) incubated with PMS alone remained its original pink color (Tube II), indicating very limited oxidative capability of PMS without activation. In contrast, RB aqueous solution transformed into colorless instantly by PMS/ZIF‐67 cotreatment, demonstrating the strong oxidative capability of PMS/ZIF‐67‐based AONP. Considering the coexistence of SO_4_^−∙^ and OH∙ in this system, quenching test was carried out to figure out which radical play the dominant oxidative role. It has been well reported that tert‐butyl alcohol (TBA) can quench OH∙, while ethanol usually reacts with both SO_4_^−∙^ and OH∙.^[^ ^15^ ^]^ As shown in Figure 2d, ethanol (tube V) but not TBA (tube IV) largely affect the oxidative capability of PMS/ZIF‐67 toward RB, suggesting that the advanced oxidation performance of PMS/ZIF‐67 system is mainly attributed to SO_4_^−∙^.

**Figure 3:**
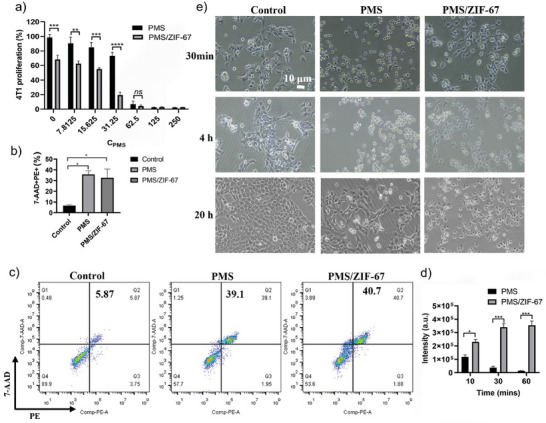
Cytotoxicity and intracellular ROS inducing capability of AONP strategy. a) 4T1 cell viability after 24 h treatment of different formulations at various concentrations of PMS. The ZIF‐67 concentration is fixed at 12.5 µg mL^−1^ (*n* = 4). b) 7‐AAD/Annexin V‐PE double positive proportion of cells after 4 h treatment (*n* = 3). c) Flow cytometry evaluation of cell apoptosis by 7‐AAD/Annexin V‐PE double staining. d) Time‐dependent intracellular ROS level of 4T1 cell treated by ZIF‐67 (12.5 µg mL^−1^)/PMS (62.5 µg mL^−1^) (*n* = 3). e) Optical images of 4T1 cell after different treatments at various time points. Scale bar: 10 µm. Statistics: one‐way ANOVA with Bonferroni's correction for multiple comparisons. All tests were two‐sided. Data were shown as mean ± SEM. *p < 0.05, **p < 0.01, ***p < 0.001, ****p < 0.0001; ns: no significant difference.

We then investigated the cytotoxicity of different treatments toward 4T1 breast cancer cells. As shown in **Figure** **3**a, despite that PMS/ZIF‐67 system and PMS exhibited a similar concentration‐dependent antiproliferation behavior, the former showed much higher toxicity than the latter under various PMS concentrations below 62.5 µg mL^−1^, suggesting that the activation of PMS by ZIF‐67 can greatly enhance its cytotoxicity. This could be explained by a higher intracellular ROS level induced by PMS/ZIF‐67 mediated AONP (Figure S4, Supporting Information), which is in accordance with the higher SO_4_^−∙^ generating efficiency of PMS/ZIF‐67 system. When PMS concentrations are above 62.5 µg mL^−1^, both PMS/ZIF‐67 and PMS groups showed significant antiproliferation activity toward 4T1 cells (<20%). To further explore the anticancer activity, 4T1 cells after treatment were costained with Annexin V‐PE and 7‐AAD and analyzed using flow cytometry. The results showed that the proportions of Annexin V‐PE and 7‐AAD double‐positive cells in PMS and PMS/ZIF‐67 groups (PMS 62.5 µg mL^−1^) were greatly increased (Figure 3b,c), while ZIF‐67 group did not induce obvious cell death compared to control group (Figure S4a, Supporting Information), which is consistent with the live/dead cell staining assay (Figure S5, Supporting Information).

Surprisingly, despite the similar apoptotic rate, the intracellular ROS level of dying 4T1 cells treated by PMS/ZIF‐67 was greatly elevated than that induced by PMS alone, and the difference becomes greater as the processing time prolongs (Figure 3d). Afterward, the cell growth and morphologies after different treatments were monitored by light microscopy. As shown in Figure 3e, compared to stretched live 4T1 cells in control group, cells in both PMS and PMS/ZIF‐67 group clearly showed typical shrunken morphology of apoptosis after 30 min treatment. However, after 20 h incubation, a proportion of cells in PMS group recovered from apoptotic condition, while cells in PMS/ZIF‐67 group remained their dying/dead status. Collectively, these results demonstrate a robust and sustained intracellular ROS inducing capability of PMS/ZIF‐67‐based AONP strategy, which generates sufficient oxidative damage to cancer cells and thus leads to unrecoverable cell apoptosis that could not be achieved by PMS alone.

**Figure 4:**
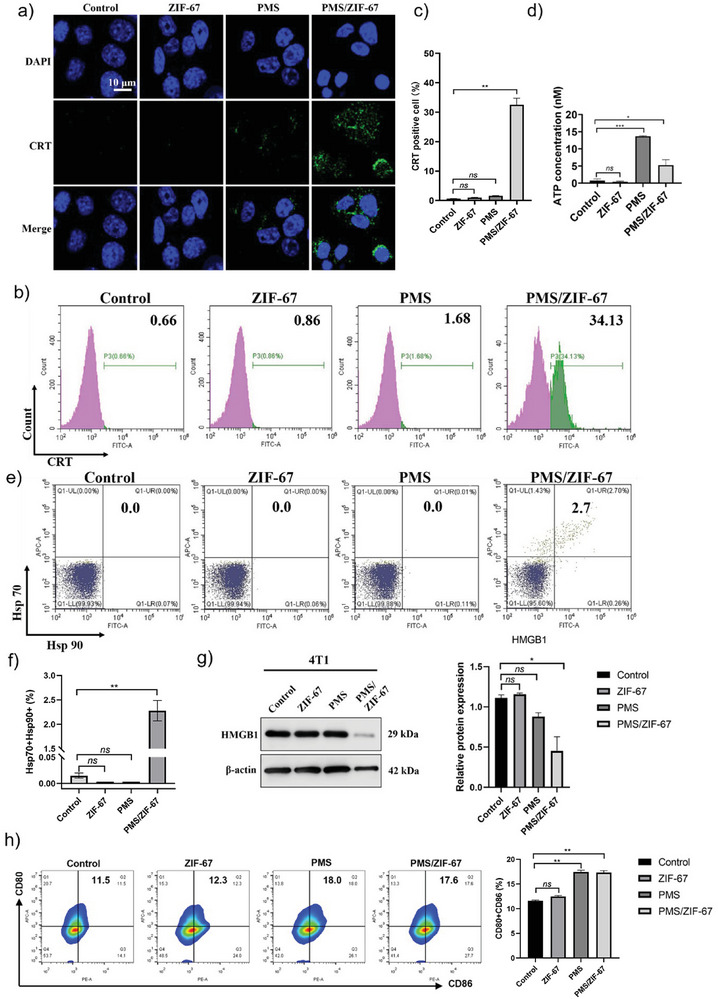
Boosted immunogenicity of dying tumor cells by AONP strategy. a) Confocal laser scanning microscopy CLSM image (scale bar: 10 µm) and b) flow cytometry analysis of the surface exposure of CRT on 4T1 cells. c) Quantitative summary of CRT positive cells based on flow cytometry analysis (*n* = 3). d) ATP release from tumor cells after treatment by different groups (*n* = 3). e,f) Expression of Hsp70 and Hsp90 quantitatively detected by flow cytometry (*n* = 3). g) Western blot analysis of HMGB‐1 from 4T1 tumor cells after treatment by different groups (*n* = 3). h) DC activation in vitro. Expression of CD80 and CD86 quantitatively detected by flow cytometry (*n* = 3). Statistics: one‐way ANOVA with Bonferroni's correction for multiple comparisons. All tests were two‐sided. Data were shown as mean ± SEM. *p < 0.05, **p < 0.01, ***p < 0.001, ****p < 0.0001; ns: no significant difference.

It is well known that the intracellular oxidative stress is a major up‐stream signal for eliciting immunogenic cell death (ICD).^[^ ^16^ ^]^ Therefore, the ICD‐inducing potential by ANOP was investigated by characterizing the exposure of DAMPs, which are considered as the hallmarks of immunogenic apoptosis.^[^ ^17^ ^]^ First, as the most typical DAMP to characterize immunogenic cell death, CRT can translocate from the endoplasmic reticulum to the cell surface under stressed condition, which can act as a “eat me” signal to stimulate the phagocytosis of dying tumor cells by antigen presenting cells (APCs).^[^ ^18^ ^]^ Analysis of CRT expression was conducted through confocal laser scanning microscopy (CLSM) (**Figure** **4**a). No surface expression of CRT in PBS or ZIF‐67 treated 4T1 cells was observed, and only negligible green signals could be observed in PMS‐treated cells. In contrast, significant CRT exposure was found on the cell surface treated by PMS/ZIF‐67. The surface exposure of CRT was further quantified by flow cytometry, showing more than ten‐fold increase of CRT positive cells than other groups (Figure 4b,c). In addition to CRT exposure, ATP is considered as a chemoattractant that acts as “find me” signals for APCs.^[^ ^19^ ^]^ Figure 4d shows that both PMS and PMS/ZIF‐67 treatment can stimulate a higher ATP release compared to control group. Moreover, heat shock protein 70

(Hsp70) and heat shock protein 90 (Hsp90) are two recently discovered “eat me” signals.^[^ ^20^ ^]^ Compared to PMS or ZIF‐67 treatment alone, only PMS/ZIF‐67 can induce the coexposure of ecto‐Hsp70 and ecto‐Hsp90 of 4T1 cells (Figure 4e,f). The migration of HMGB1 from the nucleus to cytoplasm then extracellular space is one of the DAMPs associated with ICD.^[^ ^21^ ^]^ We found that PMS/ZIF‐67 induced largely reduced intracellular level of HMGB1 (Figure 4g), suggesting the release of HMGB1 from the nucleus. Collectively, it can be concluded that PMS/ZIF‐67‐based advanced oxidation processing induced cancer cell death displaying typical biomarkers (CRT, ATP, Hsp70 and Hsp90, and HMGB1) of ICD, demonstrating their high efficiency in boosting the immunogenicity of dying tumor cells, which however cannot be achieved by either PMS or ZIF‐67 alone. The tumor‐associated antigens (TAAs) released by dying tumor cells during the process of ICD can stimulate DCs’ maturation. Therefore, we further studied the DCs’ maturation in vitro. DC2.4 cells were cocultured with 4T1 cells treated by various formulations in the Transwell system for 24 h. PMS/ZIF‐67‐treated 4T1 cells induced significantly increased expression levels of costimulatory molecules CD86 and CD80 compared to control and ZIF‐67 groups, suggesting successful induction of maturation of DC2.4 cells through AONP (Figure 4h). Interestingly, PMS groups also induced DC maturation, which is due likely to its cytotoxicity and the consequent release of certain DAMPs, such as ATP.

**Figure 5:**
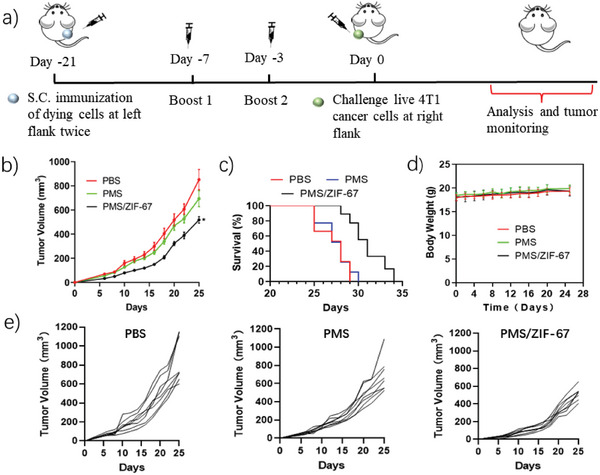
AONP boosted the immunogenicity of apoptotic cancer cells in vivo a). Schematic illustration of in vivo therapeutic protocol using PMS/ZIF‐67 catalytic system treated cancer cells to immunize mice. b,e) The overall and individual mouse tumor (left flank) growth profile of mice immunized by PBS or cells treated by different methods. c) Mice weight monitored from day 0 to day 25. d) The survival rate of mice immunized with PBS or cells treated by different methods. Statistics: data represent mean ± SEM (*n* = 8). b) Two‐way ANOVA with Tukey's correction for multiple comparisons. c) Kaplan–Meier survival analysis was used to evaluate the association between PBS, PMS and PMS/ZIF‐67. All tests were two‐sided. (*p < 0.05, **p < 0.01, and ***p < 0.001).

Encouraged by the in vitro results, a well‐established prophylactic tumor vaccination model was applied to test the immunogenicity of AONP‐treated tumor cells. Specifically, BALB/c female mice were randomly divided into three groups (namely: PBS group, PMS group, and PMS/ZIF‐67 group, *n* = 8). As shown in **Figure** **5**a, mice were immunized subcutaneously by PBS or dead/dying 4T1 cells treated by PMS or PMS/ZIF‐67 on Day −21 with twice boosts (Day −7 and Day −3) at the right flank, followed by inoculating the same type of living cancer cells on day 0 at the right flank. Due to the malignant proliferation of 4T1 cells, rapid tumor growth was observed in PBS group, and PMS treated cells exhibited very little prevention performance compared to PBS group. Encouragingly, mice immunized with PMS/ZIF‐67 treated cancer cells showed significantly delayed tumor growth profile (Figure 5b,e). The survival rate was also improved in PMS/ZIF‐67 group compared with PBS and PMS group in the 25 days after challenging with living 4T1 cells (Figure 5c). These results confirm that AONP greatly boosted the immunogenicity of apoptotic cancer cells, which successfully potentiated antitumor immune response in vivo. In addition, the body weight of all mice remained stable throughout the whole period. It should be noted that 100% and 12.5% mice were found with a tumor at the right flank upon inoculation with ZIF‐67 and PMS treated cells, respectively, as shown in the tumorigenesis test (Figures S6 and S7, Supporting Information). In contrast, mice immunized with PMS/ZIF‐67 treated cells showed no tumorigenesis. The in vivo distribution of PMS/ZIF‐67 was evaluated using fluorescent dye decorated ZIF‐67 nanoparticles. CY7 conjugated ZIF‐67 was injected subcutaneously followed by in vivo image monitoring. As shown in Figure S10a (Supporting Information), strong fluorescent signal can be observed after direct injection of CY7 conjugated ZIF‐67. In contrast, the injection of PMS/ZIF‐67 treated cells only showed very weak signal at the injection site (Figure S10b,d, Supporting Information), and no signals were found in main organs (Figure S10c, Supporting Information), evidencing that the amount of ZIF‐67 nanoparticles entering into body is negligible, which is beneficial for ensuring the biosafety of AONP strategy.

**Figure 6:**
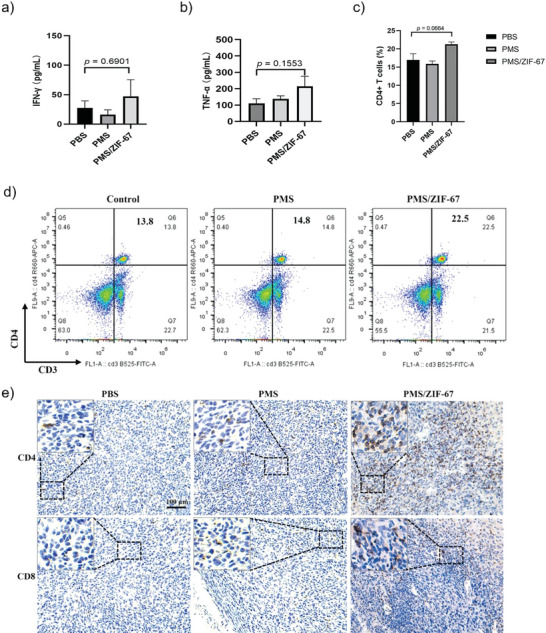
Mechanism of the AONP‐mediated antitumor immune response. a,b) Secretion of cytokines in sera measured by ELISA assay (*n* = 3). c) Quantitative summary of proportions and d) flow cytometry analysis of CD4^+^ T cells in mice spleens after different treatments (*n* = 3). e) Representative images of tumor specimens stained with anti‐CD8 and anti‐CD4 antibodies. Scale bars 100 µm. Statistics: one‐way ANOVA with Bonferroni's correction for multiple comparisons. All tests were two‐sided. Data were shown as mean ± SEM.

In order to further understand the potential mechanism of the AONP‐mediated antitumor immune response, the secretion of proinflammatory cytokines in sera and spleenocytes from immunized mice were analyzed. As shown in **Figure** **6**a,b, the highest levels of TNF‐𝛼 and IFN‐𝛾 were observed in the serum samples from mice immunized by PMS/ZIF‐67‐treated 4T1 cells, indicative of the strongest cell mediated immunity. Additionally, PMS/ZIF‐67 group also exhibited a higher average proportion of CD4^+^ T cells (CD3^+^ CD4^+^ T lymphocytes) in spleens than that in the PBS group, which is likely associated with PMS/ZIF‐67 treatment induced inflammation,^[^ ^22^ ^]^ although no statistical difference was observed (Figure 6c,d). Even not much difference was observed in the proportion of CD8^+^ T cells (CD3^+^CD8^+^ T lymphocytes) in spleen samples after different treatment (Figure S8, Supporting Information). The presence of T cells in tumors is considered to be an important biological hallmark for activation of immune response.^[^ ^23^ ^]^ Therefore, we analyzed the expression of CD4 and CD8 in the tumor of the mice on day 25. The results show that PMS/ZIF‐67 group also exhibited a higher level of intertumoral infiltration of CD4^+^ and CD8^+^ T cells than that in the PBS group as shown in Figure 6e, indicating successful activation of adaptive antitumor immunity. These results collectively support that PMS/ZIF‐67‐based AONP endows dying tumor cells with boosted immunogenicity, provoking efficient antitumor immunity and thus delaying tumor growth in vivo.

## 3 Conclusions

In summary, we have demonstrated the unprecedented application of PMS/ZIF‐67‐based advanced oxidation catalytic system in processing whole tumor cells for boosting their immunogenicity. We found that, although PMS alone could lead to comparable cytotoxicity with PMS/ZIF‐67 processing at high concentration, it failed to elicit immunogenic apoptosis of tumor cells. Only PMS/ZIF‐67 mediated AONP induced the release of multiple characteristic DAMPs, triggering efficient ICD effect. Impressively, without the assistance of any immunoadjuvants or immunotherapeutics, immunization of AONP treated whole tumor cells significantly delayed tumor growth and increased survival rate in a prophylactic vaccination model in vivo. Our AONP strategy simultaneously achieved i) well preserved antigen diversity, ii) eliminated tumorigenesis, and iii) enhanced immunogenicity of whole tumor cells.

The rationale of our developed AONP strategy encompasses four aspects: i) instead of constructing complicated drug delivery systems, this application of AONP is extremely simple and straightforward—just mixing PMS and nanocatalysts into cell culture medium; ii) PMS can be converted into non‐toxic species after activation, thus the safety risks can be largely mitigated; iii) the choice of nanosized catalysts as PMS activator ensures a catalytic process with high SO_4_^−∙^ producing efficiency and sustained oxidative stimulation toward tumor cells; iv) after treatment, the heterogeneous catalyst could easily be separated from cells by filtration, which can largely minimize the administration of nanomaterials in bodies.

Future works should focus on optimizing the particle size and composition of nanocatalysts in this system, which have been reported as important parameters in improving the advanced oxidation catalytic activity and thereby may enhance the immune stimulating effect of this system.^[^ ^24^ ^]^ Moreover, the combination of immunoadjuvants (e.g., CpG) and immune checkpoint blockades, such as PD‐1/PD‐L1 antibody with whole tumor cell antigens prepared in this work is worth of further investigation for optimal vaccination outcomes. Given the encouraging results on the advanced oxidation processing‐boosted immunogenicity of breast cancer cells, it is expected that our strategy would be adapted broadly for other types of tumor cells and pave the way to develop effective personalized vaccines.

## 4 Experimental Section

### Animal Experiments

All mice were handled in accordance with the guidelines of the Animal Care Ethics Commission of Shanghai Tenth People's Hospital and School of Medicine, Tongji University. (Code SHDSYY‐2021‐46191).

### In vivo tumorigenicity study

First, 4T1 cells were treated with ZIF‐67, PMS and PMS/ZIF‐67 respectively. Subsequently, 5 × 10^5^ treated 4T1 cells were injected into the right flank of 6‐week‐old female BALB/c mice (*n* = 8) on day 0. The size of tumors was measured every other day and the tumor volume was calculated using the formula *V* = (width)^2^ × (length)/2.

### In Vivo Vaccination with Dying Cells

The BALB/c female mice were randomly divided into three groups (*n* = 8) named as “PBS,” “PMS” and “PMS/ZIF‐67,” representing mice immunized with PBS or 4T1 cancer cells treated by PMS and PMS/ZIF‐67, respectively. Specifically, mice in “PMS” and “PMS/ZIF‐67” group were immunized with dying 4T1 cells (containing 5 ×10^5^ cells in 100 µL PBS) on day −21, −7, and −3 by subcutaneous injection into mouse right flank for three times. As a control, PBS instead of cancer cells was subcutaneously injected into the mice. On day 0, all the mice in three groups were inoculated with 5 ×10^5^ live 4T1 cancer cells into the right flank. After that, the tumor growth (*n* = 8) was monitored by measuring the tumor volumes (*V* = *W* ^2^ × *L*/2, where *W* is the width and *L* the length of the tumor). Moreover, the survival rates were also recorded throughout the whole study. In order to understand the underlying mechanism of the advanced oxidation treatment mediated antitumor immune response, blood was collected on day 7 and the serum was used for measuring TNF‐*α* and IFN‐*γ* using Elisa kit. In addition, spleens were harvested from the mice and single cell suspensions prepared by mechanical grinding. After that, red blood cells (RBCs) in the cell suspensions were removed by RBC lysis buffer, followed by costaining with anti‐CD3‐FITC, anti‐CD4‐APC, and anti‐CD8a‐PE/Cy7 antibodies and then analyzed by flow cytometry immediately.

### Immunohistochemistry Staining

Prophylactic tumor vaccination model to test the immunogenicity of AONP‐treated tumor cells. Tumor sections of each group were further studied by immumohistochemical staining assay of CD4 and CD8 on day 25. Immunohistochemistry was analyzed using standard methods. Tumor specimens were embedded, deparaffinized, hydrated, stained, and scanned.

### In Vivo Imaging Assay for Assessing the Distribution of PMS/ZIF‐67

First, ZIF‐67 was labeled with CY7 fluorescent dye. Then, AONP‐treated 4T1 cells were subcutaneously injected into the BALB/c mice. The fluorescence intensity was observed by in vivo animal imaging. After 3 days, live 4T1 cells were inoculated into the other side. Seven days later, the fluorescence signals of the inoculation site and the tumor were monitored again. Upon sacrifice, the tumors and organs were collected and imaged.

### Statistical Analysis

To ensure statistical analysis accuracy, data normalization was performed on Western blot results. The data were then expressed as relative expression levels or ratios to control for variations in protein loading and exposure time. All statistical analyses were performed with the GraphPad Prism version 8.0. All statistics were presented as the mean standard error of mean (SEM). Normality of the data was tested using the Shapiro–Wilk normality test and the Kolmogorov–Smirnov test. For two‐group comparison, *p* values were derived from the unpaired Student *t* test to determine differences between groups with normally distributed data. For multigroup comparison, one‐way ANOVA (continuous variables) with Bonferroni's post‐test or Tukey's correction for multiple comparisons were analyzed as described in the figure legends. A two‐way ANOVA (analysis of variance) was performed in the present study to examine the effects of two independent variables. All experiments were performed at least three biological replicates. All tests were two sided, and *p* values <0.05 were considered statistically significant (the *α* value was set to 0.05).

## Conflict of Interest

The authors declare no conflict of interest.

## Supporting information

Supporting Information

## Data Availability

The data that support the findings of this study are available from the corresponding author upon reasonable request.
