# Supplementary material for: Advanced Oxidation Nanoprocessing Boosts Immunogenicity of Whole Tumor Cells
Source: Adv Sci (Weinh). 2023 May 21;10(22):2302250. doi: 10.1002/advs.202302250 (PMC10401122; doi:10.1002/advs.202302250)
Supplement: Supplementary file 1 — Supporting Information [file ADVS-10-2302250-s001.pdf]

ADVANCED SCIENCE
Open Access

Supporting Information

for *Adv. Sci.*, DOI 10.1002/advs.202302250

Advanced Oxidation Nanoprocessing Boosts Immunogenicity of Whole Tumor Cells

Min Zhang, Yiming Huang, Jie Zou, Yang Yang, Yue Yao, Guofeng Cheng* and Yannan Yang**

Supporting Information

Advanced Oxidation Nano-processing Boosts Immunogenicity of Whole Tumor Cells

Min Zhang,[ǂ] * Yiming Huang,[ǂ] Jie Zou,[ǂ] Yang Yang, Yue Yao, Guofeng Cheng,* Yannan Yang*

Synthesis and PEGylation of ZIF-67: $Co(NO_3)_2 \cdot 6H_2O$ (118 mg) and PVP (100 mg) were dissolved in 10 mL of methanol to form solution A. 2-methylimidazole (133 mg) and triethylamine (4.5 μL) were dissolved in another 10 mL of methanol to form solution B. Then, solution B was added into solution A under magnetic stirring for10 min and aged for 24 h at room temperature. The products were collected by centrifugation and vacuum dry. To prepare PEGylated ZIF-67, 5 mg of PEG-NH2 (MW 5k) was dissolved in a solution containing 1.75 mL ethanol and 750 μL H_2O to make a stock solution with PEG concentration of 2 mg/mL. 5 mg of ZIF-67 were dispersed in 2.5 mL of ethanol and followed by addition of PEG stock solution and stirring overnight for PEGylation.

Materials characterizations: The morphologies of the samples were observed on a JEOL-2100F Transmission Electron Microscope (TEM) working at acceleration voltage of 200 kV. The powder X-ray diffraction (XRD) patterns were collected with Rigaku Ultimate IV diffractometer using Cu Kα1 radiation (λ = 1.54056 Å) at 40 KV and 40 mA. The free radical species were identified by Electronic Paramagnetic Resonance (EPR) tests, the measurements were performed with a Bruker EMXplus-6/1 instrument using microwave frequency of 9.82 GHz and modulation amplitude of 1.0 G, 5-dimethyl-1-pyrroline N-oxide (DMPO) was used as a spin-trapping agent. The covalent state of Cobalt in ZIF-67 was determined by X-ray Photon-electron Spectroscopy (XPS) using model Thermo Scientific *K*-Alpha equipped with Al *Kα* (*hv*=1486.6 eV) under 12 kV, the binding energy was corrected with C1s (284.8 eV). The particle size of samples was evaluated by Dynamic Light Scattering Particle Size

Distribution Analysis (DLS) using Malvern Zetasizer Nano ZS90 using ultrapure water as dispersant at 25.0°C.

In Vitro $SO_4^{-\bullet}$ verification: The advanced oxidation activity and $SO_4^{-\bullet}$ verification assay was carried out in 1.5 mL tubes, with aquoueous solution conating Rodamibne B (RB), RB+PMS, RB+PMS+ZIF-67, RB+PMS+ZIF-67+TMB and RB+PMS+ZIF-67+Ethanol, respectively. Pink color was photographed at 5 min and UV-vis-NIR spectrophotometer were used to record the absorbance of those pink supernatant at 554 nm. Notably, all operations were done at room temperature under dark. In addition, the Electron paramagnetic resonance (EPR) spectra detected by mixing 0.5 mg of the ZIF-67 and 15 mg PMS in a 50 mM DMPO solution with 5 mL aqueous dispersion for DMPO-$OH^{\bullet}$ and DMPO- $SO_4^{-\bullet}$.

Cell viability: The cell viability was measured using MTT ((3-(4,5-Dimethylthiazol-2-yl)-2,5-diphenyltetrazolium bromide) assay. Briefly, 4T1 cancer cells were seeded in 96-well plates at a density of 8,000 cells per well over night. Next day, cells were treated PMS and PMS/ZIF-67 at various concentrations of PMS. After 24 h incubation, the cell viability was determined. Cell treated with normal saline were applied as the control group and all experiments were performed in triplicate.

Apoptosis and Live/Dead Cell Staining Assay: For apoptosis analysis**,** 4T1 cells were seeded in 6-well plates at a density of 2×10^5 cells per well over night, followed by treatment with PMS (62.5μg/mL), or PMS (62.5μg/mL) /ZIF-67 (12.5μg/mL). Cells treated with normal saline were applied as the control group. After 4h incubation, cells were harvested using trypsin and washed with PBS. Then, Annexin V-PE and 7-AAD were added for cell staining and flow cytometry analysis. Instead of harvesting cells after different treatments, live/dead cell staining assay were conducted by washing and staining the treated cells with Calcein-AM/PI mixed dyes, and then imaged by an inverted fluorescence microscope at 490 nm for excitation.

ROS generation assay: ROS generation assay was assessed by plate reader and fluorescence microscope using DCFH-DA staining kit. Briefly, 8000 4T1 cells were seeded in 96-well black and clear bottom plates, followed by incubation with 10 μM of DCFH-DA (diluted from 10 mM DCFH-DA stock solution in Dimethyl sulfoxide) in serum free RPMI1640 medium for 30 min. Afterwards, unloaded DCFH-DA probe was removed and cells were treated by normal saline, PMS (62.5μg/mL) and PMS (62.5μg/mL) /ZIF-67 (12.5μg/mL), respectively. Time-dependent fluorescence intensity was measured by microplate reader at OD. For fluorescence imaging, 4T1 cells were plated into 6-well plates and incubated with DCFH-DA for 30 min. After different treatments, 4T1 cells were imaged using an inverted fluorescence microscope (Excitation: 488 nm and Emission: 505-545 nm).

In vitro ecto-CRT, ecto-Hsp70 and ecto-Hsp90 detection: CRT exposure induced by various formulations was evaluated by flow cytometry and confocal laser scanning microscopy (CLSM). For flow cytometry analysis, 4T1 cells were treated with ZIF-67 nanoparticles (12.5 μg/mL), PMS (62.5 μg/mL), or PMS/ZIF-67 (62.5 μg/mL and 12.5 μg/mL, respectively) for 4 h. Cells treated with normal saline were applied as the control group. Then the cells were washed and incubated with Alexa Fluor 488-CRT antibody for 0.5 h before analyzed by flow cytometer. For CLSM analysis, cells were incubated with Alexa Fluor 488-CRT antibody for 0.5 h, fixed with Paraformaldehyde (0.4%), stained with DAPI, and observed under CLSM using 405 nm and 488 nm lasers for visualizing nuclei and CRT exposure on the cell membrane, respectively. For ecto-Hsp70 and ecto-Hsp90 detection, the protocol was similar to ecto-CRT detection, except that Alexa Fluor 488-Hsp90 (1/1000 dilution) and Alexa Fluor 647-Hsp70 (1/1000 dilution) were used for staining.

ATP release: For ATP release test, cells were seeded into 6-well plates at the density of 2×10^5 cells/per well and incubated for 24 h. Then the original culture media was removed and the cells were treated with ZIF-67 nanoparticles (12.5 μg/mL), PMS (62.5

μg/mL), or PMS/ZIF-67 (62.5 μg/mL and 12.5 μg/mL, respectively) for 3 h. Cells treated with normal saline were applied as the control group. Cell supernatant was collected and tested in ATP Luminescence Assay Kit (A22066, ThermoFisher) following the manufacture's protocol. The luminescence was measured by a microplate reader (Synergy Mx, BioTeK).

HMGB1 detections: For release of HMGB1 analysis, 4T1 cells were treated with ZIF-67 nanoparticles (12.5 μg/mL), PMS (62.5 μg/mL), or PMS/ZIF-67 (62.5 μg/mL and 12.5 μg/mL, respectively) for 12 h. Cells treated with normal saline were applied as the control group. Then the cells were collected and tested by western blot analysis.

In vitro dendritic cell stimulation: Firstly, Residues of 4T1 cells after different treated with PMS and PMS/ZIF-67 were added into DC culture using a Transwell system. After various treatments, DCs were stained with anti-CD86-PE, and anti-CD80-APC (BioLegend), and then evaluated by flow cytometry (BD Canto II).

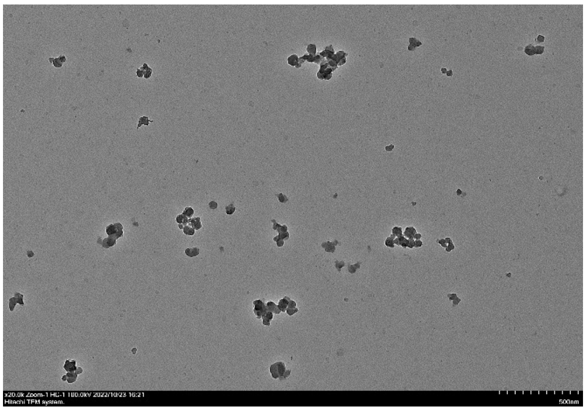

Figure S1 TEM image of ZIF-67 nanoparticles. Scale bar 100 nm.

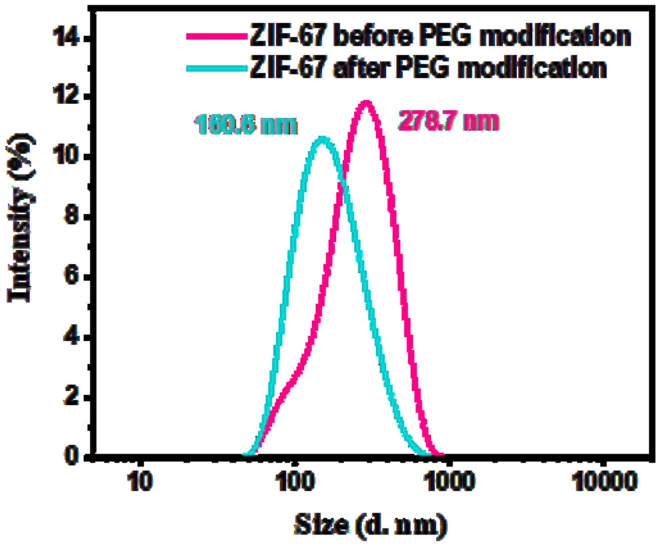

Figure S2 Size distribution of ZIF-67 before and after PEG modification dispersed in water.

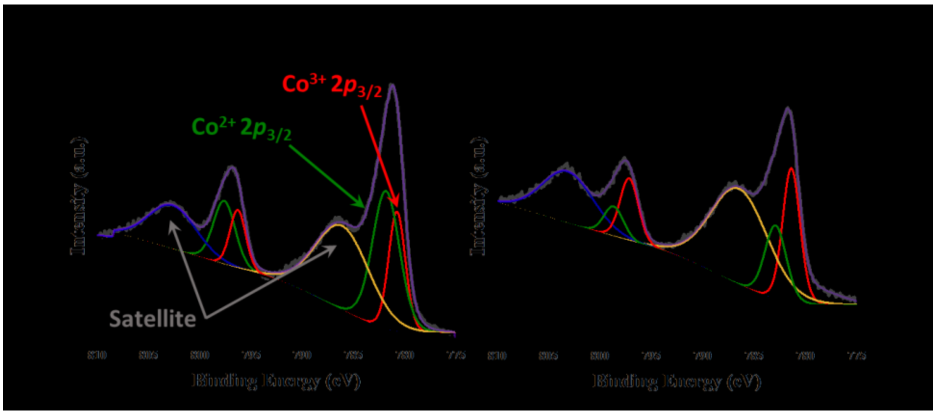

Figure S3 Co 2*p* spectra of as-prepared ZIF-67 nanoparticles (a) and ZIF-67 after PMS activation (b).

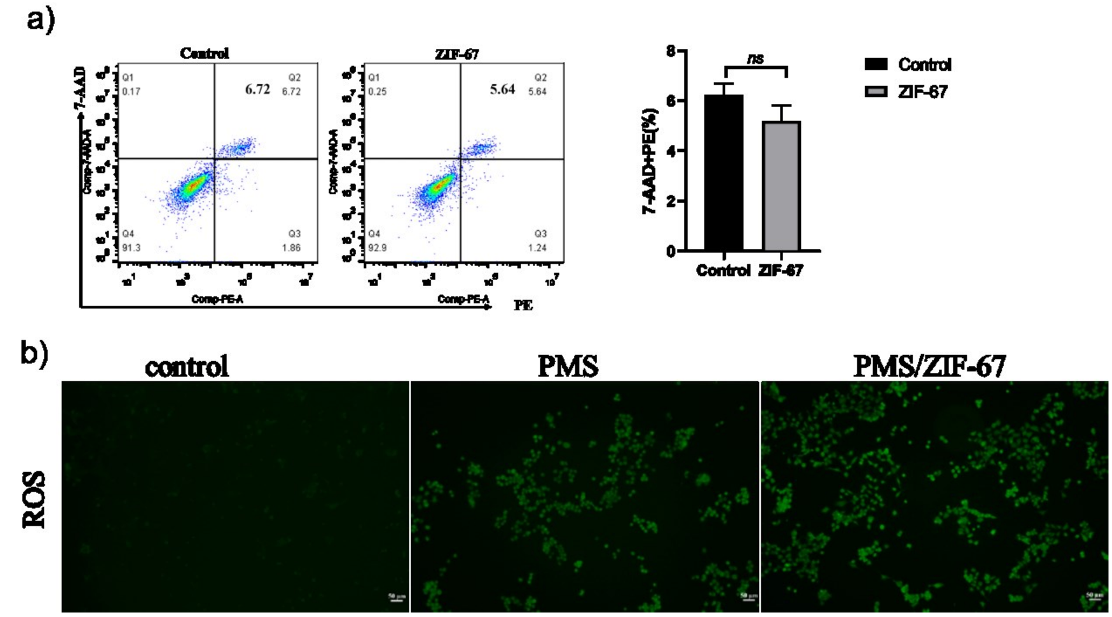

Figure S4 (a) Cytotoxicity of AONP strategy. Flow cytometry evaluation of cell apoptosis by 7-AAD/ Annexin V-PE double staining (n = 3). (b) Intracellular ROS level of 4T1 cell treated by ZIF-67 (12.5 μg/mL) + PMS (31.25 μg/mL) with DCF as fluorescence probe. Data were shown as mean ± SEM. *$p < 0.05$, **$p < 0.01$, ***$p < 0.001$, ****$p < 0.0001$, *ns*: no significant difference. Statistical analysis was performed using unpaired *t* test.

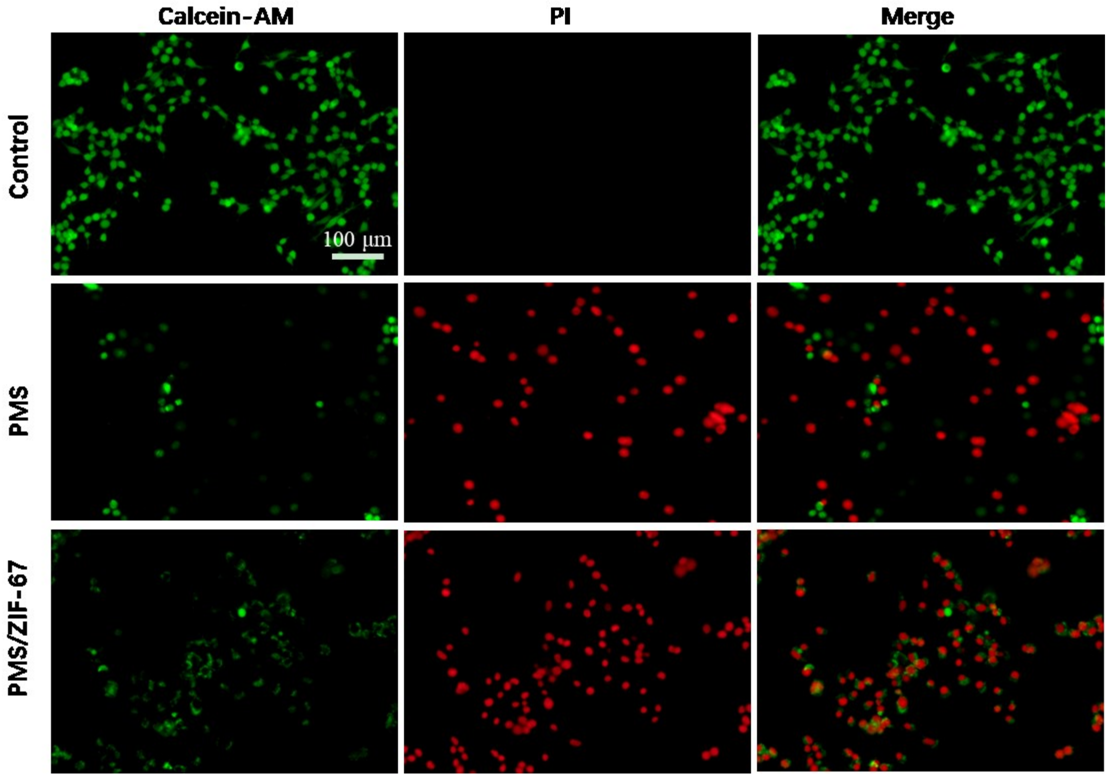

Figure S5 Evaluation of cell viability by Calcein-AM/PI double staining. The living cells were stained green with Calcein-AM and the dead cells were stained red with PI. Scale bar: 100 μm.

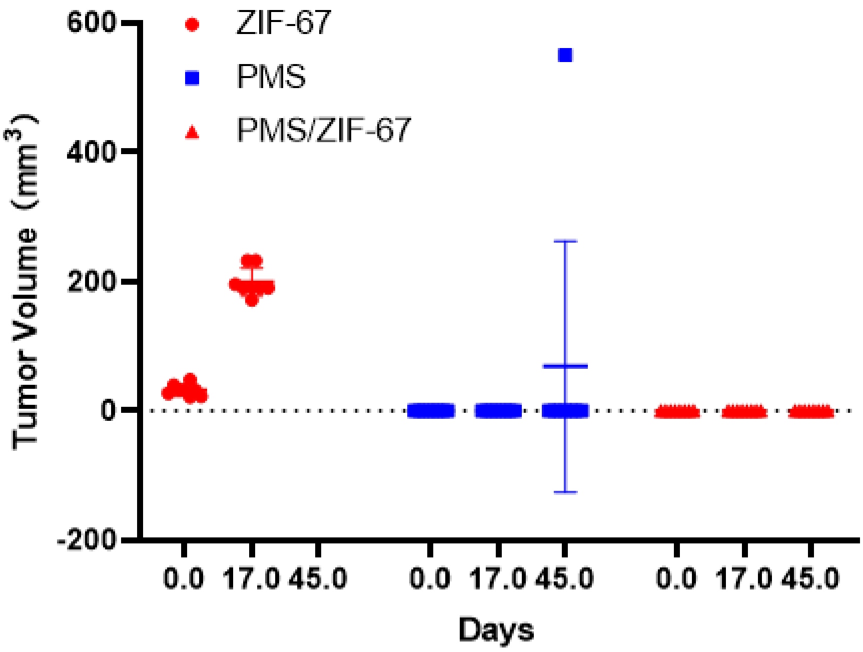

Figure S6 Tumorigenesis at right flank of mice immunized with ZIF-67, PMS, PMS/ZIF-67 treated 4T1 cells.

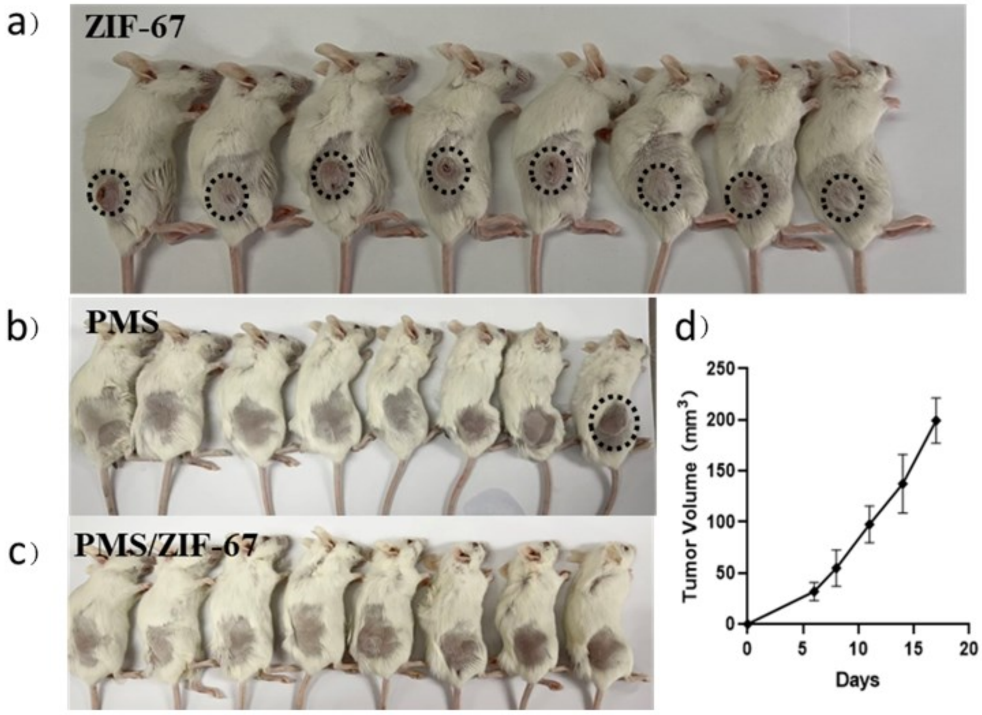

Figure S7 (a-c) Optical images of tumor formed in mice inoculated with 4T1 cells treated by ZIF-67 (day 18), PMS and PMS/ZIF-67 (day 45) at right flank. (d) The overall tumor growth profile in ZIF-67 group.

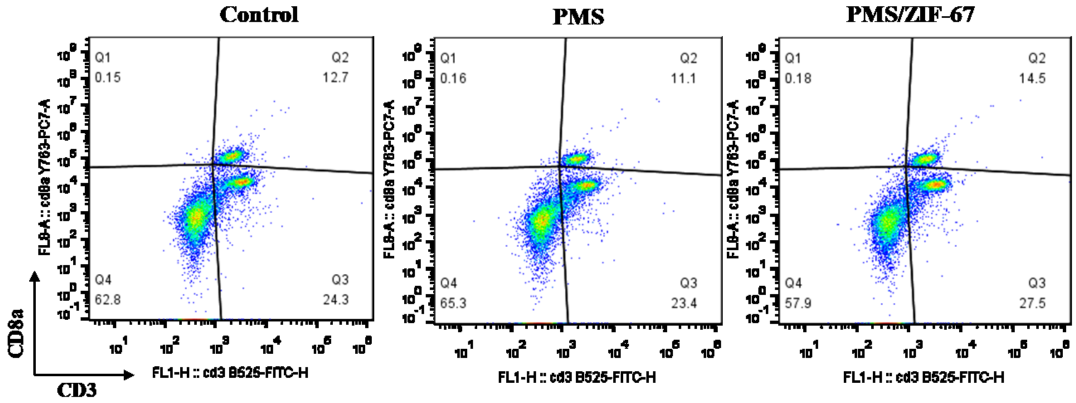

Figure S8 Populations of CD8+ T cells in mice spleens after different treatments.

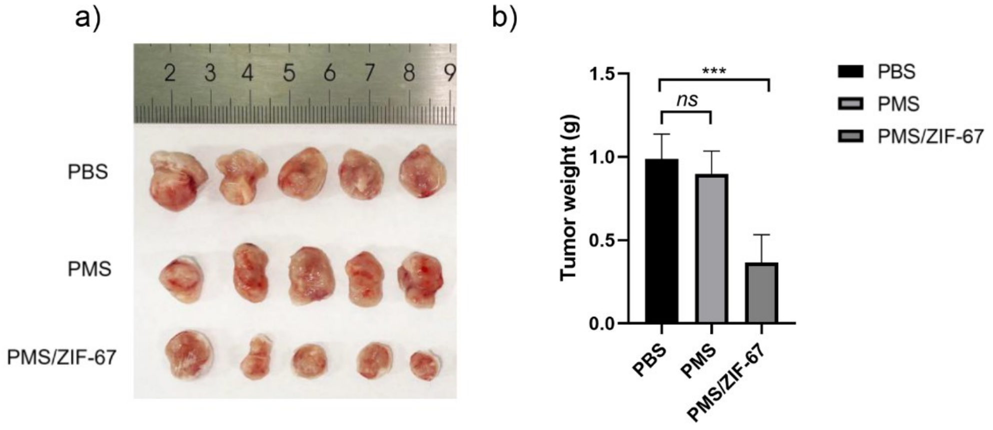

Figure S9 Prophylactic tumor vaccination model to test the immunogenicity of AONP-treated tumor cells. a) The optical images of tumors collected on day 25 (n = 5). b) Tumor weight measured. Statistics: One-way ANOVA with Bonferroni's correction for multiple comparisons. All tests were two-sided. Data were shown as mean ± SEM. *$p < 0.05$, **$p < 0.01$, ***$p < 0.001$, ****$p < 0.0001$, *ns*: no significant difference.

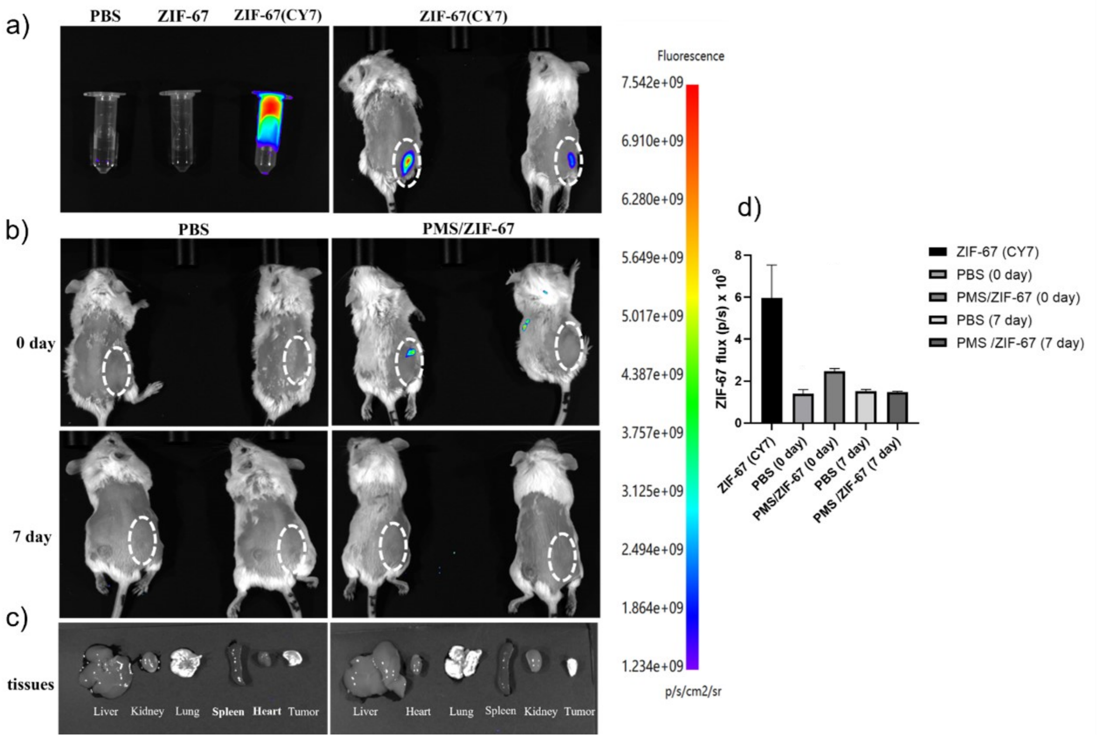

Figure S10 The in-vivo imaging assay for assessing the distribution of PMS/ZIF-67 in tumor or other major tissues. a) Fluorescence images of ZIF-67 labelled with CY7. b) In vivo fluorescence images on day 0 and 7. c) Fluorescence images of tumor and major tissues on day 7. d) Mean fluorescence intensity value of each group (n = 2). Data were shown as mean ± SEM.
